# Standardization of myocardial T1 time measurements in clinical setting using MOLLI, shMOLLI and LL at 1.5T and 3T - the CONSEPT study

**DOI:** 10.1186/1532-429X-15-S1-P18

**Published:** 2013-01-30

**Authors:** Toby Rogers, Darius Dabir, Tobias Voigt, Tobias Schaeffter, Eike Nagel, Valentina O Puntmann

**Affiliations:** 1 Cardiovascular Imaging, King's College London, London, UK; 2 Philips Innovative Technologies, Clinical Research, London, UK; 3 Department of biophysics and medical engineering, King's College London, London, UK

## Background

MMeasurement of myocardial T1 mapping is possible with most of the clinically used magnets using sequences based on modification (MOLLI) or shortening (shMOLLI) of the Look-Locker (LL) sequence. However, the robustness of T1 post-processing approaches has not been systematically examined for these sequences, nor for different field strengths, in native and post-contrast T1 maps. Moreover, repeatability of measurements in conditions with altered geometric relations of cardiac chambers and wall thickness commonly observed in clinical settings remains unknown.

## Methods

**Figure 1:**
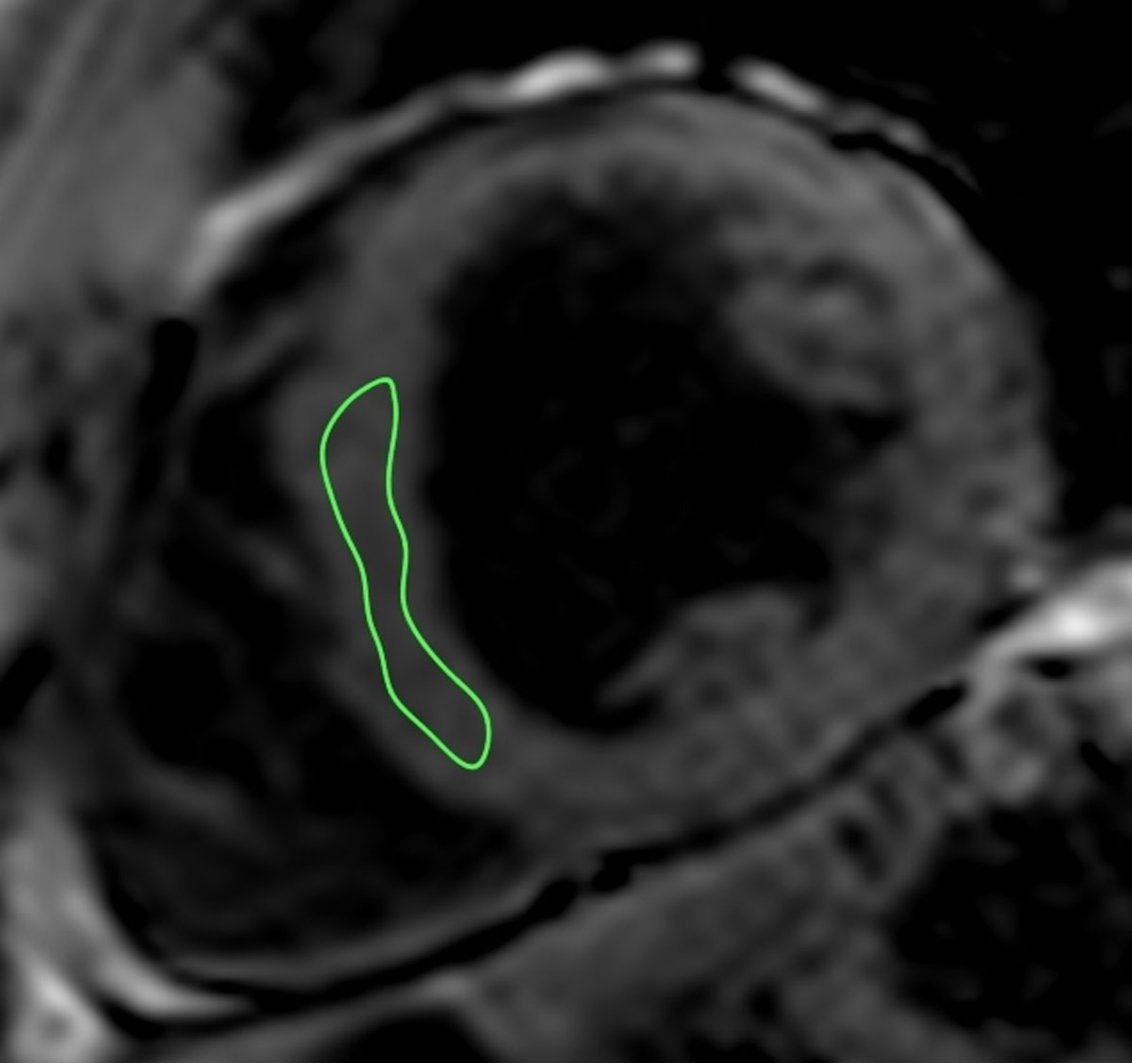
The CONSEPT technique: a region of interest (ROI) is plotted conservatively within the septum well away from the myocardial-blood pool interface. The ROI is then semi-automatically propagated across all images in the sequence and manually corrected for cardiac and respiratory motion.

We evaluated T1 maps obtained in 82 consecutive subjects (age 51.2 ± 14.8; male n=53) referred for clinical cardiac magnetic resonance (CMR). On the basis of CMR findings we created 3 groups with respective field strength (1.5T, 3T) subgroups: increased LV wall thickness (IVSd>12 mm, LVPWd>11 mm; n=6, 9), and increased LV chamber (EDV/BSA> 100 ml/m^2^; n=11, 26). Fifteen subjects with normal CMR findings and low pretest likelihood served as controls (n=15,15). T1 maps were acquired in mid-ventricular short axis pre- and post-contrast using MOLLI, shMOLLI and LL sequences. Two independent observers drew ROIs conservatively within the septal and lateral myocardium, followed by semi-automatic propagation through the phases and manual correction for cardiac and respiratory motion (the CONServative SEPTal or CONSEPT technique - see Figure1). In patients with ischaemic scar, infarct areas confirmed on late gadolinium enhancement images were excluded from the ROI measurements.

**Figure 2:**
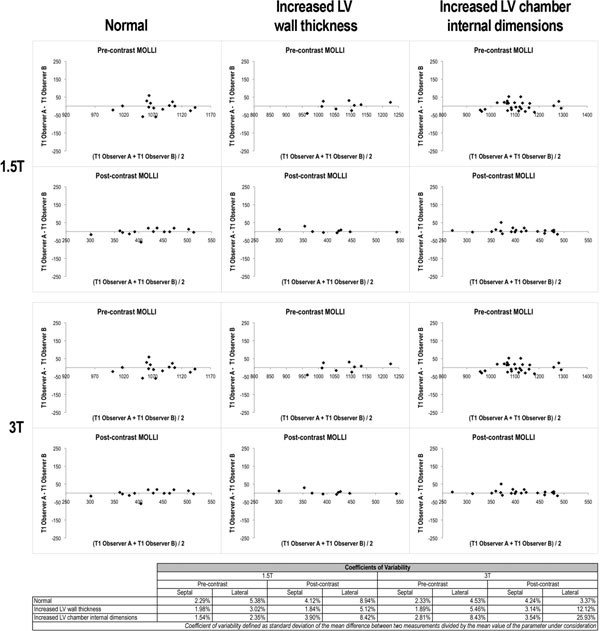
Bland-Altman plots demonstrating excellent inter-observer repeatability of T1 time measurements using the CONSEPT technique and coefficients of variability demonstrating wider variability in the lateral wall compared with the septum.

## Results

T1 times derived in septal myocardium showed excellent intra and inter-observer repeatability at both field strengths and in all diagnosis categories (CoV=1.5- 4.1). In contrast, lateral wall T1 time measurements showed considerably wider inter-observer variability (CoV=2.4-9). In addition, the CoVs were significantly higher in at 1.5T field strength, shMOLLI, and LL sequence (p<0.05). Regionally reduced signal-to noise ratio, sampling error from voxels straddling the myocardial-blood pool interface and measurement error in the lateral wall underlined the greater inter-observer variability in the lateral wall compared with the septum.

## Conclusions

We demonstrate that measurement of T1 values using conservative septal technique (CONSEPT) is robust and repeatable in healthy subjects and also in patients with significantly altered chamber relations. We propose the CONSEPT approach as the standardized post-processing method for T1 value derivation in clinical setting.

## Funding

National Institute for Health Research (NIHR) comprehensive Biomedical Research Centre award

